# Understanding the self-management experiences of people with heart failure with preserved ejection fraction (HFpEF), their caregivers and the health care professionals who support them: Systematic review and qualitative meta-study

**DOI:** 10.12968/bjca.2024.0084

**Published:** 2025-06-02

**Authors:** Julia Frost, Samantha Barbara van Beurden, Jess C Bollen, Valerie Wells, Colin Greaves, Chim Lang, Rod Taylor

**Affiliations:** Health and Community Sciences, University of Exeter Medical School, South Cloisters, University of Exeter, St Luke's Campus, Heavitree Road, Exeter, EX1 2LU, UK; Health and Community Sciences, University of Exeter Medical School, South Cloisters, University of Exeter, St Luke's Campus, Heavitree Road, Exeter, EX1 2LU, UK; Health and Community Sciences, University of Exeter Medical School, South Cloisters, University of Exeter, St Luke's Campus, Heavitree Road, Exeter, EX1 2LU, UK; MRC/CSO Social and Public Health Sciences Unit, School of Health and Wellbeing, University of Glasgow, Clarice Pears Building, 90 Byres Road, Glasgow, G12 8TB; School of Sport, Exercise & Rehabilitation Sciences, University of Birmingham, Edgbaston, B15 2TT; Division of Molecular & Clinical Medicine, School of Medicine, University of Dundee, DD1 9SY; MRC/CSO Social and Public Health Sciences Unit, School of Health and Wellbeing, University of Glasgow, Clarice Pears Building, 90 Byres Road, Glasgow, G12 8TB

**Keywords:** Heart failure with preserved ejection fraction (HFpEF), Meta-study, Qualitative synthesis, Secondary Analysis, Self-management, Systematic review

## Abstract

**Aims:**

To understand the self-management experiences of people with heart failure with preserved ejection fraction (HFpEF), their caregivers and the health care professionals (HCPs) who support them.

**Methods and Results:**

A systematic review of qualitative and mixed methods literature, published to July 2023. 4560 abstracts describing patients, caregivers, and HCPs experiences of self-management strategies for HFpEF were identified and screened. Of the 349 full texts identified we included seven papers from three data sources (in 47 patients, 81 caregivers, 129 HCPs) in our final dataset. Key theories, methods and results were extracted, reviewed and synthesised using meta-study techniques.

We identified that HFpEF is difficult for HCPs to diagnose and difficult for patients and their caregivers to understand. Patients and caregivers can struggle to access support, which can lead to a ‘cycle of decline’ over time in patients’ outcomes /quality of life. However, self-management can be optimised with adequate support, when the burdens of understanding HFpEF and self-management are acknowledged, when personalised goals are set, and feedback is provided to optimise individualised self-management.

**Conclusion:**

The limited capacity of HCPs to support HFpEF patients and caregivers to develop effective self-management understanding and practice can lead to disempowerment and loss of control. Tailored interventions are required to maximise HCP enablement of patients and their caregivers to develop flexible and sustainable self-management strategies. Future research must specify and evaluate the skillset that HCPs require to provide individualised care that is tailored to the holistic needs of people self-managing HFpEF within the local care context.

## Background

The prevalence of heart failure (HF) globally is estimated at 38 million patients (1). HF constitutes 10% of the total NHS and comparable global budgets (3; 4); with hospitalisation accounting for 80% of the total costs (5). Heart failure with preserved ejection fraction (HFpEF) accounts for half of all HF cases (6). While HF is associated with poor health-related quality of life; HFpEF is specifically associated with reduced ability to engage in activities of daily living, reduced exercise capacity, poor health-related quality of life (HRQoL), high rates of hospitalisation, and premature mortality (3; 11; 12).

We have previously been involved in the development, evaluation, and roll-out of the Rehabilitation EnAblement in Chronic Heart Failure (REACH-HF) intervention, a facilitated home-based cardiac rehabilitation programme designed to optimise self-care for adults with heart failure with reduced ejection fraction (HFrEF) (13). We are currently evaluating the effectiveness of the adaptation of the REACH-HF intervention for HFpEF.

The aim of this paper is to understand the self-management experiences of people with HFpEF and their caregivers, and the perspectives of the HCPs who support them.

### Research questions

1. What are the experiences of self-management for adults with HFpEF and their caregivers?
2. What are HCPs’ perspectives of facilitating self-management programmes for people with HFpEF and their caregivers?

## Methods

We employed meta-study to appraise and synthesise the qualitative literature (16). Meta-study was developed to synthesise the findings of existing research by interrogating the philosophical underpinnings of the study, and how decisions are made in the design and conduct of the research, to inform the research product (17; 18). It involves four processes:

- *meta-data-analysis*: explores the empirical findings in the primary research papers;
- *meta-method*: assesses the epistemological validity and rigour of the research methods employed;
- *meta-theory*: examines the philosophical approach underpinning the original research; and
- *meta-synthesis*: consolidates the three steps and considers the plausibility of existing accounts, any gaps in knowledge, and the scope for advancing new knowledge (19).

Here we use meta-study in a diagnostic sense to identify what the current state of knowledge is, and in a reflexive sense to inform future research.

A scoping exercise established that it was difficult to identify papers explicitly about lay experiences of HFpEF (*Question 1*). This was due to challenges around the definition of HFpEF, and ways in which some papers used a generic definition of HF. We used the SPIDER tool for qualitative evidence synthesis to develop a broad search strategy (23; 24), which an information specialist [author] ran in April 2022 and updated in July 2023. (Supplementary File 1):

*Sample:* People with HFpEF; Caregivers;

*Phenomenon of Interest:* Usual care; Rehabilitation.

*Design:* Published literature of any research design, grey literature.

*Evaluation*: Characteristics, views, experiences

*Research type:* Qualitative (using a filter developed for qualitative research (24), quantitative and mixed methods peer-reviewed studies.)

A database search, without date restrictions, was conducted (CINAHL, EMBASE, MEDLINE, PsycINFO, Web of Science databases -Science Citation Index Expanded (SCI-EXPANDED); Social Sciences Citation Index (SSCI); Conference Proceedings Citation Index – Science (CPCI-S); Conference Proceedings Citation Index – Social Science & Humanities (CPCI-SSH); Emerging Sources) (Clarivate)). References, including titles and abstracts, were then loaded into Rayyan bibliographic software (25). Papers were included at the title and abstract screening stage using explicit eligibility criteria (Box 1):

Scoping of the topic regarding the role of HCPs (*Question 2*), employed our learning from the first search (above): that if the abstract did not include terms specifically related to HFpEF or its synonyms, then there was no discussion of HFpEF in the sample or analyses. We therefore ran a simplified search strategy about the HCPs who facilitate self-management in October 2022 and re-ran in July 2023 (Supplementary file 2). Searches were run in the same databases as the first searches (above). References, including titles and abstracts, were then loaded into Rayyan. Our eligibility criteria were identical to the first search, with ‘participants’ now HCPs. Identified titles and abstracts were screened for potential eligibility. Screening and data extraction were conducted independently by two reviewers and checked by a third [author, author, author]. Uncertainties and disagreements were resolved by discussion between the team. Full text papers of all potentially includable studies were obtained and screened independently for inclusion in the review. Key authors were also asked if they could suggest any other relevant papers (26).

### Data extraction

Two researchers independently extracted data from the papers using a validated meta-study template (19), to enable assessment of the rigour and validity of any theoretical frameworks informing the research design, data collection, and analytical techniques used by the research authors, to inform our synthesis (Table 1).

The same researchers independently applied the Critical Appraisal Skills Programme (CASP) checklist to assess the quality of the studies selected for inclusion (27) (Table 2), which further supplemented the data extraction process and facilitated our understanding of the nature of the primary papers (29).

### Data analysis

Meta-study aims to provide a systematic means for both analysing and synthesising research literature to confer meaning on the findings and to construct a more extensive basis for understanding the phenomena of interest (19). We commenced with *meta-theory* (18), because in the included papers theoretical approaches to qualitative research *informed* analyses, even when applied post-hoc.

## Results

**Figure 1:**
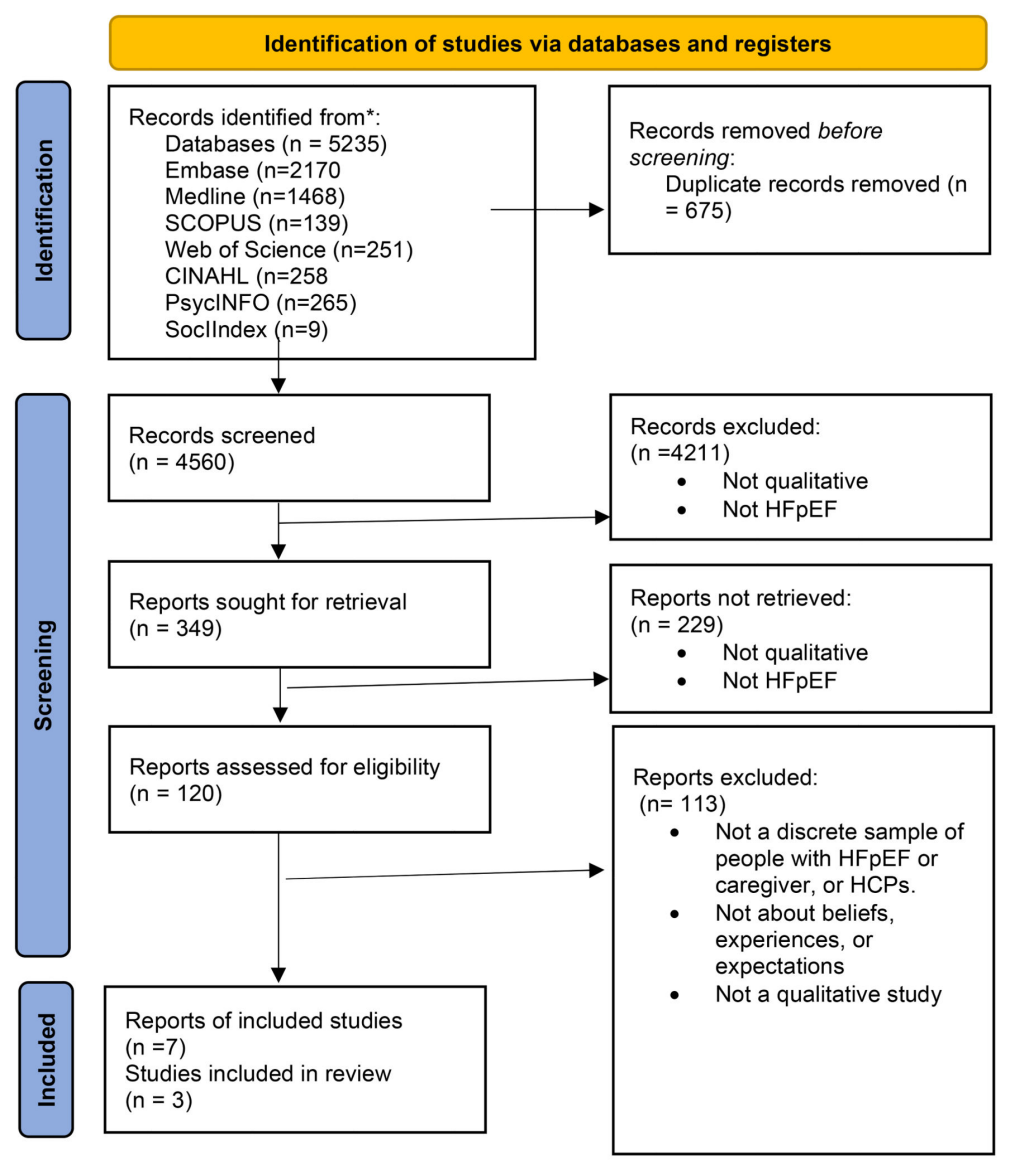
PRISMA 2020 flow diagram for: Understanding the self-management experiences of people with heart failure with preserved ejection fraction, their caregivers and the health care professionals (HCPs) who support them.

The searches resulted in 4560 abstracts (Figure 1). Preliminary screening identified that 307 abstracts detailed qualitative papers describing patients’, caregivers’ and HCPS’ experiences of self-management strategies for HFpEF. A full text screen identified six papers (30–35), while direct author contact identified a further paper in press (36). This dataset comprised interviews or focus groups with 47 patients, 81 caregivers, and 129 HCPs.

### Meta-theory

The seven papers report on three studies, with each study having a particular focus:

- focus groups with HCPs, oriented to diagnostic uncertainty (30);
- interviews with patients, caregivers and HCPs, to understand and maximise clinical decision-making and management in primary care (31, 32, 34–36);
- interviews with patients and caregivers, to evaluate whether a self-help intervention could be adapted for people with HFpEF (33).

The disciplinary perspectives of the authors framed the articulation of the research questions and research methods employed. The earliest paper was conducted by a multi-disciplinary team from the Northeast of England (30), who employed a hermeneutic phenomenological framework (37; 38) to update and extend an earlier qualitative study which had identified barriers and facilitators to HF management (39). They (30) included a range of specialist and non-specialist HCPs to explore their decision-making, beliefs, and experiences regarding “what can and should be done” (30: 2).

Five of the papers were outputs from the Optimise HFpEF research programme, led by a Professor of Nursing, and a multi-disciplinary team, collecting data from three locations in England (the east of England, Greater Manchester, and the West Midlands; 31). The objective of the study was to inform the development of an optimised programme to address the needs and experiences of people with HFpEF and healthcare providers (40). These papers involved primary data analysis (31; 32), secondary analysis (35; 36), and both (34). The application of new theories in subsequent papers, align with the intentions of the authors to investigate new research questions and verify existing findings to extend the understanding of patient and HCP needs (41).

Chronologically, the first of the Optimise HFpEF y papers employed Normalisation Process Theory (42) post-hoc to “provide sensitising constructs” to assist interpretation and “guide recommendations” (31:e881). The second paper was concerned with the management of HFpEF by GPs in primary care, with a focus on the ‘challenges encountered by GPs’ associated with managing HFpEF in people from Black, Asian and Minority Ethnic (BAME) backgrounds (32). The third sought to explore the role and experiences of informal carers of people with HFpEF, because of the perceived complexity of their role. The authors hypothesised that the challenges experienced by carers would be accentuated in HFpEF due to limited knowledge and understanding among formal healthcare providers (34). The fourth paper undertook a secondary analysis of the patient and caregiver interviews, applying the Cumulative Complexity Model (43) as a lens to explore the causal processes of HFpEF patients’ deterioration (35). Contacting the study authors identified a written patient perspective on the third paper (44), and a fifth paper: a secondary analysis of interviews alongside analysis of guidelines that employed ‘soft systems methodology within the critical realist paradigm’ (45) to interrogate the difference between any HFpEF ‘*work-as-imagined’* in discourse and *‘work-as-done’* in practice (36).

In the paper undertaken as part of a process evaluation of a pilot study of the REACH-HF intervention (14) adapted for HFpEF (33), a pragmatic approach was used to assess the fidelity of intervention delivery and patients’ and caregivers’ experiences of participation in the intervention.

We identified that current research has sought to develop theory from the data (30; 32; 34) or apply mid-range theories retrospectively, to inform future intervention development (31; 35; 36).

### Meta-method

During screening, we identified that the experiences of HFpEF were rarely reported explicitly but often aggregated into a more generic ‘HF experience’. Thus, many papers were excluded from our synthesis because we could not identify a sub-sample of HFpEF, or a threshold from which we could extract HFpEF cases from a mixed HF sample.

The earliest included paper sought to capture change in service delivery and management of HF over time, and undertook focus groups with GPs, GP partners, cardiologists, general physicians and HF nurses recruited from health authority registers (30). Purposive sampling enabled “a diverse representation of gender, role, seniority, ethnicity, geographical distribution, employment status (part or full time) and practice size (group or single-handed) and avoided over-representation of individual practices” 30: 2). To stimulate discussion during the focus groups, participants were presented with clinical vignettes, which included the diagnosis but not the management of HFpEF specifically.

The Optimise HFpEF study was designed to interrogate the needs of patients, caregivers and HCPs both cross-sectionally and longitudinally (40). Various members of the Optimise HFpEF research team shared data and were involved in all subsequent secondary analyses, to extend questions or answer new ones (46).

Sowden et al (31) undertook interviews and focus groups across three geographical areas of England, recruiting HCPs and patients from primary and secondary care, employing purposive sampling to ensure variance in patient and HCP characteristics. Topic guides were designed to capture patient, caregiver and HCP perspectives of living with and managing HFpEF.

The focus of the research conducted by Hossain et al (32) was GP experiences of managing people with HFpEF in primary care, and explicitly those from BAME backgrounds; however, no information is provided about how GPs working with BAME patients were sampled for or recruited, and none of the questions in the topic guide addressed the needs of people from BAME backgrounds.

Pearson et al (34) explored the role and experiences of informal carers of people with HFpEF, by purposively recalling caregivers from earlier studies in the HFpEF research programme, and exploring practical tasks associated with caregiving and whether these aspects were viewed as challenging or rewarding. These interviews were combined with existing data from the research programme, although earlier interviewers were not “specifically targeted at carers and caregiving experiences, but more broadly explored the HFpEF experience” (34: 3).

In the fourth paper, Forsyth et al (35) undertook a secondary analysis of interviews previously conducted with patients, carers and HCPs, although no information is provided about the basis on which interviews were sampled in the new interpretation.

Finally, Brooman-White et al (36) sampled 25 interviews from the ‘Optimise HFpEF data corpus’ (comprising a total of 50 patients, 9 carers/relatives and 73 clinician interviews), although the authors state that “further interviews were sampled, after determining initial themes, to seek multiple perspectives… adequate information power and examine referential adequacy” (36). However, no information is provided about the additional sample, nor whether these interviews had been sampled in any of the prior publications from the study. Although the Optimise team employed ‘auto-data’ to maximise the reach of the data collected, sometimes the rationale for, or the composition of the sample was unclear, which inhibited the extent to which data extraction and appraisal could identify whether the ‘sorting and sifting’ processes of secondary analysis were appropriate and rigorous (47).

Smith et al (33) undertook an assessment of the fidelity of intervention delivery by listening to and scoring audio recordings of intervention delivery sessions provided by a trained HCP (a nurse trained in CR, and another in HF) following an a priori checklist of items relating to the style of delivery and intervention components to be delivered. Thematic analysis of interviews with patients and caregivers was also undertaken to assess their experiences of participation in the REACH-HF intervention (33).

Across the papers much methodological information was provided about the research design and data collection procedures; however, prioritisation was given to descriptions of sampling processes, with less attention paid to the exploration of negative cases (e.g. where patient experiences deviated from or refuted the majority experience) (19).

### Meta-data analysis

In the earliest paper, the research team demonstrated inertia in diagnosis and effective management, and ambiguity around diagnostic criteria and effective treatment options - for patients and all HCP groups (30).

The first of the five papers in the Optimise HFpEF programme analysed all of the interviews and focus groups from the three geographical areas, using Framework Analysis and Normalisation Theory to identify tensions between lay and professional accounts of how HFpEF is identified and understood (31). Results are framed in relation to ‘management disparity’ when compared with HFrEF, such that: “access to cardiac rehabilitation or exercise programmes was variable; other cardiac or pulmonary conditions appeared to be prioritised.” (31: e885) An identification of the ‘misattribution’ of symptoms resulted from the limited capacity of HCPs to support and educate people with HFpEF, and a reliance on family members and patients to “coordinate care, which was problematic when a clear understanding of their heart failure was lacking” (31: e886).

The second paper analysed data collected from GPs using Framework Analysis four themes were generated from the data, with all themes “considered more challenging when managing people with cultural and language differences” (32: 6). While challenges associated with managing BAME patients are mentioned in relation to them being more likely to have comorbidities, language differences, and lifestyle, the needs of people from BAME backgrounds are not addressed in the conclusion, nor solutions to mitigate these challenges proffered.

The third paper analysed patient and caregiver interviews using thematic analysis, which produced a typology of care provided and care relationships: labelled as supportive, instrumental, and reciprocal (34). The results are presented using the analogy of a performer “keeping plates spinning” (34) although it is not clear if the analogy was developed from the data, the researchers, or existing literature. The paper concludes that the responsibilities and roles of caregivers are complicated by the lack of recognition and understanding about HFpEF, which inhibits access to resources, such as HCPs with HFpEF expertise.

The fourth paper was conducted as a secondary analysis of the patient and caregiver interviews, using Thematic Analysis, which the authors suggest enabled the identified relationships and interactions in the data to be aligned with the cumulative complexity model (35). This alignment led the authors to conclude that spiralling complexity occurs when physical, social and psychological decline inhibit a person’s ability to self-manage HFpEF, which leads to a sense of disempowerment and loss of control:

“so that patients have the physical and psychological capacity they need to prevent the cycle of decline described.” (35: 7)

These findings are underscored by an associated patient think piece, which emphasises the importance of collaboration and personalised goal setting:

“You may come across articles and professional advice that propose ‘there is no therapy for HFpEF’, which discourages clinicians, patients and carers. But this does not have to be. By working together with our GP, getting to grips with self-monitoring and building strong self-care routines, we have learnt to live our best life, in spite of HFpEF.” (44:e14)

In the paper by Brooman-White et al (36), by juxtaposing discourse analysis with interview analysis, the authors established the ‘distance’ between the ‘imaginary’ of the guidelines and the ‘reality’ of clinical practice, enabling a ‘space’ for ‘the design of interventions to improve coordination of care’ which could include strategies for accommodating local conditions, while facilitating multi-disciplinary working (36).

Finally, Smith et al (33) analysed recordings of an intervention delivered by HCPs using a scoring system to establish fidelity to the intervention delivery model, and thematic analysis of subsequent interviews with patients and carers (33). Findings suggest that HFpEF patient and their carers have unmet needs that the intervention, originally developed for HFrEF, has the potential to meet - but to do so requires facilitators adept at motivational interviewing and goal setting not only with patients but also their informal caregivers who are crucial to successful self-management (33). The requirement for the presence of this skill set was not reported in the other papers.

Tabulating the data enabled us to identify that analytical attention has focused on the practices of the HCPs, with relatively little attention on patients’ self-management practices or what their specific self-care needs might be (19). There were also limited examples of the papers building on each other’s findings (19), although most were conducted in the last few years.

### Meta-synthesis

We looked across the papers for participant data and author explanations (also called ‘first’ and ‘second’ order data) to identify salient concepts within the body of literature as a whole (also called ‘third order’ concepts (50)) (Table 4). By applying the three prior processes we were able to develop a coherent line of argument which suggests that:

HFpEF is difficult for HCPs to diagnose and difficult for patients and their caregivers to understand. Patients and caregivers can struggle to access both a diagnosis and adequate support, which are made more challenging by organisational barriers between HCPs. This can make self-management challenging and lead to a ‘cycle of decline’ over time in patients’ outcomes /quality of life (35). However, HFpEF self-management can be optimised with adequate support, when the burdens of understanding HFpEF and self-management are acknowledged, when personalised goals are set, and feedback is provided to optimise individualised self-management.

## Discussion

We conducted a meta-synthesis to consider the plausibility of existing accounts, any gaps in knowledge, and the scope for advancing new knowledge (19). The primary data contains examples of HCP, patient, and caregiver uncertainty; but that self-management can be optimised with appropriate support, when the ‘work’ of self-management is acknowledged, and when personalised goals are set, and timely feedback provided (33; 36). These papers confirm, rather than extend, our understanding of how patients and caregivers experience self-management of HFpEF (51–53). Future research needs to specify and evaluate the skillset that HCPs require to provide individualised care that is tailored to the holistic needs of the individual and the local care context.

The studies synthesised are cross-disciplinary and informed by mid-range theories of intervention development and evaluation. With such a relatively young field of study, the papers tend to be descriptive or exploratory, rather than being explanatory, which could inhibit more interpretive or normative considerations (19). However, the papers from the OPTIMISE HFpEF and REACH-HF programmes recognise that tailored interventions are required to maximise the enablement of HFpEF patients and their caregivers to develop flexible self-management strategies (55).

This paper has employed lesser-utilised qualitative research methodologies (meta-study and secondary analysis) to better understand a poorly understood condition (HFpEF). This has enabled us to identify that people with HFpEF struggle to be diagnosed, which in turn can make it harder for them to be identified as potential research participants.

### Strengths and Limitations

The use of meta-study to ensure rigour in our synthetic practice. To ensure that our results are credible and trustworthy, we have presented data from the primary reports, used comparable descriptions and interpretations as in the primary research, to produce conclusions that are grounded in the research processes and analytical steps (19). By synthesising research conducted with over 250 research participants, we can better understand how the organisational and relational challenges (e.g. discontinuity in clinical management) directly impact HFpEF self-management. By re-using, rather than replicating, previous research (63) we have been able to identify the interplay between systemic, organisational and individual factor that inhibit effective HFpEF self-management, and sites for future solutions. We recognise that our work is one interpretation, and the researchers from other disciplinary backgrounds, using other synthesis methods could reach other conclusions (19). A limitation is that the three studies were conducted in the UK, and future research needs to identify variation in international patient and HCP perspectives and self-care support practices.

## Conclusion

The limited capacity of HCPs to support HFpEF patients and caregivers to develop effective self-management understanding and practice can lead to disempowerment and loss of control. Patient and caregiver perspectives suggest that tailored interventions are required to maximise HCP enablement of patients and their caregivers to develop flexible and sustainable self-management strategies. Future research is needed to specify and evaluate the skillset that HCPs require to provide individualised care that is tailored to the holistic needs of people self-managing HFpEF within the local care context.

## Supplementary Material

Supplementary Material

## Figures and Tables

**Table 1:** Study characteristics Key: (S) sample from Sowden paper, (F) sample in Forsyth study.

Author Year Country	Objectives	Recruitment/Sample	Theory	Methods	Data Analysis	Results about self-management specifically Conclusions
**Focus groups and a national online cross-sectional survey**						
Hancock et al 2014 (30) England	To explore challenges in HCPs views about diagnosis and managemen t of heart failure since a study in 2003	(HCPs) Focus group participants were recruited from health authority registers from the North East of England, including salaried GPs and GP partners, cardiologists, general physicians and HF nurses.	(AP) A hermeneutic phenomenologi cal Framework (*cites Husserl 1970; Moustakas 1994*)	Focus groups (n=8 with a total of 56 participants) with clinical vignettes covering the diagnosis including HFpEF. Themes derived from the focus groups informed the development of a UK survey which sought to assess views about care provision from diagnosis to the end of life.	Thematic data analysis conducted by two phenomenologists. Findings were subsequently discussed by the whole team. (*cites Husserl 1970; Moustakas 1994)* Themes derived from the focus groups informed the development of a UK survey which sought to assess views about care provision from diagnosis to the end of life.	5 themes emerged: *The diagnostic process* *Diagnostic tests* *The value of clinical guidelines* *Tensions between the individual and organisational practice.* *Uncertainty about end of life care* **Discussion:** Of note was the fact that 9% of cardiologists remained unconvinced about the existence of HFpEF, which may have influenced the confidence of other clinicians when considering a diagnosis of HFpEF.
**OPTIMISE HFpEF cohort**						
Sowden et al 2020 (31) England	To explore clinicians’ and patients’/carers’ perspectives and experiences about the managemen t of HFpEF to inform the developmen t of an improved model of care	[Patient (S) + Caregivers (S) +GPs (S)] Purposive sampling strategy aimed to ensure variability in age, sex, and comorbidities in patient participants; and a range of organisational practices and clinicians involved in managing care (GPs, practice nurses, HF specialist nurses, and cardiologists). Patients with diagnosed or suspected HFpEF were eligible for inclusion unless they were identified by the direct care team as having cognitive impairment, being non-English speaking, receiving end-of-life care, or having another life-threatening condition. Research sites were identified across the east of England, Greater Manchester, and the West Midlands, with the support of the National Institute for Health Research Clinical Research Network. *(Cites Ritchie and Lewis 2014)* A total of 50 patients, nine carers/relatives, and 73 clinicians were recruited from 26 GP practices and nine hospitals from across five NHS trusts	(PH) Framework analysis and Normalisation Process Theory [once data analysis commenced]. *(Cites Ritchie and Lewis 2014; and May et al 2020)*	Interviews and focus groups were conducted by five of the authors, all of whom have training and experience in qualitative methods; two were non-clinicians and none had a previous relationship with participants. Written consent was obtained from all participants. Conversations were digitally recorded, transcribed verbatim, and checked for accuracy of transcription by a researcher before analysis. Reflexive notes were recorded by researchers throughout the process. Caregivers and relatives were interviewed as dyads when accompanying patients at the interview or at a patient’s request.	The analytical approach occurred in two phases: Framework analysis was used to identify key patterns in the data relating to the research objective, and Normalisation process theory (NPT) was used to provide sensitising constructs to reframe and further interpret the findings and guide recommendations. As this was an exploratory study, applying a theoretical framework too early in the formal analytical process could have imposed preconceptions and assumptions on the data. Therefore, researchers remained grounded in the data during the first phase of analysis, which involved iterative stages… Joint coding and discussions about the coding and interpretations took place at regular team meetings (face-to-face and by telephone). Analytical themes were also reviewed by investigators from the wider research programme and a patient advisory group to ensure that findings were credible and confirmable. Patient and clinician frameworks were initially developed separately, but as the data were analysed iteratively during the charting and interpretation process, the coding frame was expanded, refined, and combined to identify key overarching barriers to the optimal care of patients with HFpEF. In the second phase, the explanatory model, NPT informed an evaluative view of the themes identified through framework analysis. *(Cites Ritchie and Lewis 2014; and May et al 2020)*	Three main themes: *Diagnostic difficulty.* *Unclear illness perceptions.* *Management disparity.* **Implications for research and practice** This study illustrates the uncertainty and variability surrounding the management of HFpEF. The NPT construct of coherence may help explain how a lack of shared understanding and identification of this condition had the potential to influence how care was enacted, coordinated, and appraised. More work is required to raise the public and clinical profile of HFpEF, to ensure widespread differentiation and awareness of this condition. The development of a clear set of accepted practices that assimilate well-defined roles and responsibilities in its management also has the potential to improve care. Furthermore, equitable provision of services is required, as is the development of systems that improve access and integration across primary and secondary care settings. Consequently, there is a pressing need for the development of interventional research aimed at ensuring optimal practice underpinned by coherence, thus legitimising approaches to primary care management of the growing population of patients with HFpEF. Continued collaboration of key stakeholders will be essential in the development and design of future interventions.
Hossain et al 2021 (32) England	To explore the perspectives of GPs on the identificatio n and managemen t of people, including those from ethnic minority groups, with Heart Failure with Preserved Ejection Fraction (HFpEF).	[GPs (S)] GP participants were recruited from general practices within three regions in England (the North West, East of England and the West Midlands), to ensure sampling of practices working with different systems and providing care in a range of areas, to different populations… Participants were recruited with support from the National Institute for Health Research Clinical Research Networks (NIHR CRN). Purposive sampling was used to identify and select the GP participants by criteria, such as gender, a range of years of experience in general practice, partnership status (GP partners, salaried, and locums). A sample of 35 GPs from across the three study regions were recruited from 19 practices	N/A	Data were collected through semi-structured interviews (n = 35) and focus groups (n = 1). Interviews were conducted either face-to-face at a place of the participant’s choosing (usually their practice) or by telephone, by MH, ES and IW (experienced health service researchers) between October 2017 and July 2019. One focus group with eight GPs was conducted (facilitated by CD & IW), as a pragmatic approach at one general practice, for convenience and to enable an interactive discussion among the GPs… Interviews and focus group followed the same topic guide which provided a frame of reference, rather than an inflexible structured process. *(Cited Braun and Clarke 2019)*	Framework analysis was used to analyse the data – a matrix-based format that facilitated sharing data as a team. Although focus groups and individual interviews had been conducted by a team of research associates (MH, ES, IW), all team members were involved in analysis as a team by regular team meetings (face-to-face and by telephone), with discussion of the coding and codebook. All research associates kept a research diary to record their reflective notes during the interviews and thoughts about analysis throughout the process. Familiarization involved researcher carefully read and reread the interview transcripts, their reflective notes and re-listening all the interviews. Initially, two researchers from each region independently coded the first few transcripts and circulated the codebook to the researchers of all three regions. The [Patient Advisory Group] was also involved in reviewing the analytical themes and offered alternative viewpoints to ensure themes were reliable and self-evident…Researchers then applied the final analytical framework to each transcript using the QSR NVivo Software. *(Cited Ritchie and Lewis 2013).*	Themes presented reflect four interrelated challenges: *GPs’ lack of understanding of HFpEF, which complicated* *difficulties in communicating the diagnosis, leading to* *uncertainty regarding patient management, further hindered by* *discontinuity across the primary/secondary care interface.* **Implications for clinical practice** It is important to raise awareness and knowledge about HFpEF to enable GPs to consider and make the diagnosis and support the management of patients in primary care. This will allow for a recognition of the complexity of the condition and provision of personalised care where possible. Special attention is needed to support people with health literacy and language difficulties, such as people form BAME groups. Education and training are essential to increase GPs’ knowledge and skills in considering HFpEF as a possible diagnosis. Communication between primary and specialist care teams is key, with clear referral pathways established to support the management of people with HFpEF.
Pearson et al 2022 (34) England	Heart failure with preserved ejection fraction (HFpEF) accounts for 50% of all heart failure cases; yet remains poorly understood, diagnosed, and managed, which adds complexity to the carer role. No study to date has investigated the experiences of informal carers of people with HFpEF. The aim of this study was to explore the role and experiences of informal carers of people with HFpEF.	[Caregiver (S) + (F)] Patients with HFpEF and their carers derived from two separate studies conducted across three geographically dispersed regions in England (Cambridgeshire, the Midlands, and Greater Manchester). Following an amendment to existing ethical approvals (REC reference: 17/LO/2136), participants were purposively recalled from an established cohort based on documented characteristics relating to carer. Adult carers were purposively sampled and were provided information about the study while they accompanied participants for follow-up or by mail. For inclusion, adult carers had to be .18 years old, able to communicate in English, and self-identify as an informal carer. Interviews from a previous qualitative component (REC reference: 17/NE/0199) that included a carer were also included in this analysis. All participants provided written informed consent, and the study conformed to the Principles of the Declaration of Helsinki. Interviews were stored securely according to University of Cambridge data protection policies, and all transcripts were fully anonymized and labelled with identification number only.	N/A	Interviews were guided by a semi-structured interview schedule that developed from the literature review, expert input, and discussion with a patient and public panel. Interviews were audio-recorded with digital devices. Most interviews were performed face to face (n = 20), two were telephone interviews. Most interviews with carers were performed as dyads with patients contributing. Interview schedules did not change over time but were informed by previous responses and familiarization in an iterative process. Interview schedules are available on the study website (https://www.optimisehfpef.phpc.cam.ac.uk/study-documents/). All interviews were transcribed verbatim, anonymized, and checked against interview recordings. Interviews conducted as part of the earlier qualitative component were not specifically targeted at carers and the caring experience, but more broadly explored the HFpEF experience. Therefore, beyond consent, minimal data about these carers were obtained and descriptions of time spent caring and demographic data were drawn from interview narratives. Those purposively sampled from the cohort study (n = 12) provided more extensive quantitative data including estimated time spent providing care and detailed demographic information. All interviews were conducted by experienced healthcare researchers, two of whom (F.F. and C.D.) are clinically qualified with a specialist interest in HFpEF. Participants recruited from the cohort study had attended previously for clinical assessment, therefore there was an established rapport.	Anonymized transcripts were uploaded and managed in NVivo12. The six phases of thematic analysis as described by Braun and Clarke (2006) were followed. Familiarization (Phase 1) was performed by all researchers. Initial data-driven coding (Phase 2) of five interviews was performed independently by three researchers (C.R.P., F.F., and E.K.) and checked for consistency/variance. Codes and code descriptions based on the first pass coding were agreed and the remainder of the transcripts was coded to this frame by C.R.P. with additional codes being added as needed. The final codebook was reviewed independently by two researchers (C.P. and F.F.) who separately sorted the codes into potential themes which were visualized as thematic maps (Phase 3: searching for themes). All authors reviewed the thematic maps, agreed the candidate themes through discussion, and checked theme coherence at code and data corpus level (Phase 4: reviewing themes and Phase 5: defining and naming). During coding, no new themes were generated beyond the first 10 interviews and the additional interviews added confirmation and detail. Each narrative was interrogated to establish the type of care provided and care relationships were categorized according to a broad descriptive typology: supportive [general support excluding activities of daily living (ADLs)], instrumental (general support plus assistance with one or more ADLs), reciprocal (where both parties experience morbidity and care is reciprocal dependent on health status/need), or derived from multiple sources (a person who draws on multiple sources such as family, friends, and the wider community, where no single source was identified as providing the most assistance) (ADLs)], instrumental (general support plus assistance with one or more ADLs), reciprocal (where both parties experience morbidity and care is reciprocal dependent on health status/need), or derived from multiple sources (a person who draws on multiple sources such as family, friends, and the wider community, where no single source was identified as providing the most assistance)	Thematic analysis resulted in the generation of the analogy of a performer keeping multiple plates spinning. Three key descriptive themes characterized the dataset: *the complex nature of informal caregiving (‘spinning plates’);* *the barriers to caregiving (‘the spinning falters’); Theme 3. the facilitators of caregiving (‘keeping the plates spinning’).* **Conclusion** Carers of people with HFpEF supported their loved ones to manage many of the health and day-to-day responsibilities faced by patients living with chronic conditions. The care role described was complex and dynamic with both patients and carers exhibiting tremendous resource to maintain independence. Roles and responsibilities were complicated by structural (organizational) barriers to care, driven by an identity crisis in HFpEF (lack of recognition and understanding), which appeared to moderate access to important support resources such as a HFMDT.
Forsyth e al 2023 (35) England	To investigate how heart failure with preserved ejection fraction (HFpEF), within the context of limited clinical services, impacts patients’ lives.	[Patients (S) + (F) + Caregivers (S) + (F)] Anonymized transcripts from interviews (n=62) conducted with individuals with HFpEF (n=61, denoted a ‘P’) and their informal carers (n=16, denoted as ‘C’), **collected as part of two previous studies,7,18 were collated**. *(Cites Sowden et al 2020; Pearson et al 2022)* Patients with a confirmed diagnosis of HFpEF and their nominated carers were recruited from three geographical areas in England. Four researchers, with experience in qualitative methodology, conducted interviews.	(PH) The Cumulative Complexity Model is an evidence-grounded model which emphasizes the functional mechanisms of complexity at the level of the patient. Central to the CCM is an interactional process between two concepts: patient workload (day-to-day tasks and responsibilities) and patient capacity (ability, resource, and readiness) to address demands. Workload and capacity affect each other and affect healthcare access, use, and enaction of treatment. The model proposes that imbalances in workload and capacity and barriers and facilitators to healthcare access influence health outcomes in their own right. However, workload and capacity are further mediated through the burden of illness (the effects of diseases including physical, psychological, and socio-economic) and burden of treatment (demands placed on patients by healthcare systems) feedback loops.	Interviews, conducted either over the phone or face to face, were digitally recorded and transcribed verbatim. Topic guides, which can be viewed on the study website https://www.optimisehfpef.phpc.cam.ac.uk/, did not change but were informed by concurrent analysis. The mean interview length was 59 min and all transcripts were checked against recordings for accuracy.	A secondary thematic analysis of interview transcripts from 77 people diagnosed with HFpEF and their carers, informed by the Cumulative Complexity Model. Regardless of the original study in which patients and carers were enrolled, they were prompted to reflect on their experiences of symptoms, the process of diagnosis, and subsequent management. Transcripts were coded in NVivo 12 software by one author (F.F.). Multiple cycles of sorting and defining, in line with the phases of Thematic Analysis described by Braun and Clarke (2006) were performed. During the mapping phase, (Braun and Clarke 2006) the relationships and interactions between codes were theorized. This process highlighted similarities with an extant model, the cumulative complexity model (CCM)(Shippee 2012). The CCM offered both a lens and a framework through which to view, understand, and explain the complex interactional nature of experiences and eventual causal process of deterioration frequently described in interviews. As such, inductively derived themes were deductively mapped to the core concepts described in the CCM. *Trustworthiness* Codes were verified by another author (C.D.) via comparison with themes from previous analyses and verification against transcripts. A patient advisor (J.S.) also contributed to codes, themes, theory application, and drafts of the Manuscript.	Four themes corresponding to the core concepts of workload, capacity, access, and outcome described in the CCM were generated: *shouldering a heavy workload;* *multiple threats to capacity;* *deficient illness identity;* *spiralling complexity.* **Conclusion** Exploring the HFpEF experience through the lens of the CCM enables the generation of an explanatory model of decline in HFpEF whereby a heavy workload and multiple threats to capacity beget a poor outcome, with neither potential targets (workload or capacity) being ameliorated through presentation at healthcare services. Whilst many similarities exist between the experiences of people with HFpEF and HFrEF, particularly around burdens relating to self-care and treatment, people with HFpEF appear to experience additional barriers to comprehensive care through deficient illness identity. The visibility of HFpEF must be elevated so that it is perceived as an illness carrying importance and understood as a condition worth diagnosing and actively treating. Clinicians must also work with patients to ensure that workload-capacity difficulties are identified and improved, so that patients have the physical and psychological capacity they need to prevent the cycle of decline described.
Brooman-White et al 2023 (36) England	To explore how care is coordinated for patients with HFpEF, with a focus on the interface between primary care and specialist services in England.	[Patients and HCPs, but **? if from S or F samples**] Interview transcripts for secondary analysis were purposively sampled from within the Optimise HFpEF data corpus, which comprises interviews with 50 patients, 9 carers/relatives and 73 clinicians. Initial 25 interview samples included a range of stakeholders identified from guideline review (general practitioners (GPs), HFSNs, cardiologists, practice nurses and patients/patient and carer dyads). Further interviews were sampled, after determining initial themes, to seek multiple perspectives (as required by the STEW principles), obtain adequate information power and examine referential adequacy. In total, 41 interviews were sampled from the Optimise HFpEF data corpus (table 3). Patients (N=13) Clinicians (N=28; of which General Practitioners =11; Practice Nurse =4; Heart Failure Specialist Nurse = 5; Cardiologists =4; Other HCPs =3)	(AP) Systems thinking aids understanding of complex situations when developing and implementing improvement initiatives. One approach has been to explore and attempt to reconcile the difference between *work-as-imagined* and *work-as-done. Work-as-imagined* is how work processes may be documented in guidance and protocols. *Work-as-done* is the way people who do the job adapt working practices based on system conditions they face (such as lack of information, demand outstripping capacity or suboptimal equipment). The ‘Systems Thinking for Everyday Work’ (STEW) principles can aid understanding of complex processes representing *work-as-done* in healthcare and develop recommendatio ns for change. In this study, the STEW principles were used to understand the healthcare system experienced by patients with HFpEF with a focus on the interface between primary care and specialist services. Our objectives were to compare how guidelines describe coordination of care *(work-as-imagined)* with the experience of patients and healthcare professionals (*work-as-done*) and to use this insight to generate suggestions for change.	Interviews were collected as a component of the Optimise HFpEF Study, which aimed to explore the perspectives of patients/carers and clinicians across primary and secondary care to develop understanding of how this group can be better managed. *(Cites Forsyth 2019, Optimise protocol paper).* Researchers purposively sampled a range of stakeholders, with the intention of gaining adequate information power to form a deep understanding of multidisciplinary HFpEF care. The sampling strategy aimed to ensure variability in age, sex and comorbidities in patient participants, and a range of clinicians involved in HFpEF care. (*Cites Sowden et al 2020)* Recruitment took place between October 2017 and July 2019 across three regions (Cambridgeshire, Greater Manchester and the West Midlands). Interviews were conducted by 5 trained qualitative researchers (3 clinicians, 2 non-clinicians), including face-to-face and telephone, and were conducted as patient/carer dyads as per patient preference… Further details regarding study procedures are included within the Optimise protocol and outputs. *(Sites Forsyth protocol paper, Sowden et al 2020 and Hossain et al 2022)*	None of the authors of this paper conducted interviews sampled for this work. The author who led on analysis for this paper (RB-W) was not involved in primary analysis of this data set. Two of the authors (TB and CD) were involved in primary analysis and topic guide development. Systems issues across the primary /secondary care interface were identified through primary analysis. As such, this secondary analysis provides a more in-depth analysis of an aspect of the data only partially addressed in the primary study. This project was developed and undertaken in keeping with soft systems methodology within the critical realist paradigm. Soft systems methodology was developed in engineering to define and tackle problematical situations. *(Cites Checkland 1990)* Healthcare systems, such as the one around patients with heart failure, involve people, processes, technology, a physical environment and other interconnected systems depending on where artificial boundaries are drawn. When considering improvement initiatives, we must consider these components and their interactions to determine how to better ‘engineer’ it. The STEW principles consider multiple perspectives of real-life, everyday work. The STEW principles are used in this study as a sensitising framework for thematic analysis of the data to explore how and why everyday work differs from work as imagined in guidelines. STEW principles direct the exploration of how work conditions (such as demand/capacity issues, availability of resources and constraints), interactions and flow of work and competing goals result in people adapting the way they work. These adaptions manifest as trade-offs, workarounds and result in variability of care which may or may not be beneficial in the care of patients with HFpEF. Through exploration, they provide a framework for considering practical resolutions to problems.	*3 themes were identified:* *working with complexity,* *information transfer and* *working relationships.* **Working with complexity:** Patient descriptions of their route to diagnosis were often circuitous, for example, following several different speciality appointments… Patients described managing multiple, diverse appointments, frequently in the context of poor mobility, frailty and limited transportation options. **Information Transfer:** Patients generally valued being copied into letters. However, some felt that these lacked understandable information, particularly regarding medications and diagnoses, which could cause distress. *“It’s just the terminology they use really. I mean, if [patient1] received this letter on his own, he would think, what’s that? That* *sounds horrible. What does that mean? (P1 carer)* **Working relationships:** Patients with accessible, relational continuity with a clinician reported a feeling of cohesion around their care, compared with patients who struggled to obtain this. *So even a nurse practitioner at the practice, whose keeping an overall view of what’s going on and what’s happening would help… there isn’t anybody… it Just all seems very random.* (P2) **Discussion:** Patients in this study described working to coordinate their own care. Indeed, encouraging patient self-management is promoted in policy and guidance, and has been associated with reduced healthcare utilisation. Our results confirmed primary analysis findings that self-care capacity is dependent on knowledge and access to health service support. Recent literature suggests patient work may be a helpful and necessary source of system resilience. **Recommendations:** Support patient education to enable understanding of individualised decisions in complex long terms conditions such as HFpEF. Enable patient engagement through provision of accessible support, for example, a key contact. **Conclusion:** Our findings have implications, which could affect the design of interventions to improve coordination of care. More widely, aligned with policy and research, they reflect the importance of acknowledging system complexity as we strive to improve care quality along-side increasing medical and social multimorbidity.
**REACH-HFpEF pilot trial**						
Smith et al 2021 (33) Scotland	This paper presents the process evaluation sub-study of the REACH-HFpEF pilot trial that sought to assess the fidelity of intervention delivery and patients’ and caregivers’ experiences of participation in the REACH-HF intervention.	(HCPs, Patients + Caregivers) Between April 2015 and June 2016, 50 patients were randomised (intervention group, n = 25; control group, n =25). Patients had a mean (± SD) age of 71 (± 10.7) years with a similar proportion of men and women. Caregivers were typically a spouse or partner, younger (mean age 66 ± 10.6 years), and female. The process evaluation sample was representative of the trial intervention group. Fidelity of intervention delivery The six patients and caregivers included in the fidelity analysis contributed a total of 41 facilitator interactions. Fifteen patients, seven with caregivers, were interviewed.	(AP) The process evaluation included a qualitative and quantitative assessment of both the intervention fidelity (i.e. the quality and consistency of the facilitators’ delivery of the REACH-HF intervention) and a qualitative exploration of both HFpEF patient and caregiver experiences of the REACH-HF intervention, through semi-structured interviews.	The interview assessed (1) participants’ understanding of their condition, (2) engagement with the REACH-HF intervention in supporting their adjustment to daily living with HFpEF, and (3) the perceived benefit of the intervention, including self-care behaviours and coping skills. Participants were encouraged by the researcher and through further probing to openly express their views. Interviews were audio-recorded and transcribed verbatim. Field notes were also completed (JC) to enable reflection on the process, the interviewer’s performance, and participants responses to questions and to provide contextual information to the analysis, where relevant. Reflexive memo notes were kept to assure the transparency and trustworthiness of the analysis.	Thematic analysis of the transcripts (led by JC and supported by KS) included data familiarisation through repeated listening to the audio recordings and review of interview transcripts. Initial codes, which summarised the content either descriptively or interpretively, were created. Codes with common features were then grouped together into emergent themes, before finally being assigned to three interpretive overarching themes. The themes are illustrated using participant quotes. Independent analysis of a sample of three transcripts by KS reflected the initial data codes, provisional themes, and sub-themes suggested by JC. Discussion and interpretation of these findings allowed refinement of themes/subthemes through development of definitions for each, as well as consideration and exploration of additional perspectives and explanations. All participants were asked if they wanted a copy of the interview transcript to review and add comments; none requested this. Both facilitators were also interviewed about their experience of delivering the intervention (by KS) using the same process described above. *(Cited Braun and Clarke 2006)*	Three overarching themes and related subthemes emerged from the analyses: *understanding their condition,* *emotional consequences of HFpEF, and* *response to the intervention.* **Conclusions** This process evaluation provides important evidence supporting the feasibility and acceptability of delivering the REACH-HF intervention that has the potential to address some key unmet needs in HFpEF patients and their care-givers. One of the most important intervention components identified by this study was the role of the healthcare facilitator, who acted as an educator, a source of support and reassurance, as well as a motivator and enabler. The facilitators helped to reframe participants’ thoughts to enable engagement in activity, symptom monitoring, and self-management of their HF through realistic goal setting and pacing. The study also identified how involving caregivers was at times challenging, and a more proactive strategy may be required to optimise this part of the intervention in future applications and clinical trials. The findings of this process evaluation will inform a future multicentre trial.

**Table 2:** Quality appraisal Key: Y=Yes, N=No, ?= Could not tell/Unclear

	1. Clear statement of aims	2. Appropriate methodology	3. Research Design appropriate	4. Recruitment strategy appropriate	5. Data collection address the research issue	6. Relationship between researcher and participant	7. Ethical issues taken into consideration	8. Data Analysis sufficiently rigorous	9. Clear statement of findings	10. Value of the research
Hancock et al 2014 (30)	Y	Y	?	?	?	N	N	Y	Y	Y
Snowden et al 2020 (31)	Y	Y	Y	Y	Y	N	Y	Y	Y	Y
Hossain et al 2021 (32)	Y	Y	Y	Y	Y	N	Y	Y	Y	Y
Smith et al 2021 (33)	Y	Y	Y	Y	Y	N	?	?	N	?
Pearson et al 2021 (34)	?	Y	Y	?	Y	?	Y	Y	N	Y
Forsyth et al 2023 (35)	Y	Y	Y	Y	Y	?	?	Y	N	?
Brooman-White et al 2023 (36)	Y	Y	Y	?	Y	Y	Y	Y	Y	Y

**Table 3:** Tabulation of data to identify areas that have been explored Key: AP: A priori, I: Iterative, PH: Post Hoc.

Author	Theory	Data collection	Analysis	Themes
Hancock et al 2014 (30)	Hermeneutic Phenomenological Framework (AP)	HCPs	Thematic	The diagnostic process Diagnostic tests The value of clinical guidelines Tensions between the individual and organisational practice. Uncertainty about end of life care
Snowden et al 2020 (31)	Normalisation Process Theory (PH)	Patient(S) + Caregiver (S) + GP(S)	Framework	Diagnostic difficulty, Unclear illness perceptions, Management disparity.
Hossain et al 2021 (32)	Cumulative Complexity Model (PH)	GP(S)	Framework	Lack of understanding HFpEF, Difficulties in communicating the diagnosis, Uncertainty in managing people with HFpEF, Discontinuity across the primary and secondary care interface
Smith et al 2021	Process evaluation (AP)	HCPs, Patients + Caregivers	Thematic	Loss of identity, Recognising and responding to emotion, Engagement with the REACH-HF intervention, Changes in health-related behaviours, monitoring and symptom tracking, Unique caregiver views and experience.
Forsyth et al 2023 (34)		Patient(S)+(F) + Caregivers (S)+(F)	Thematic	Shouldering a heavy workload, Multiple threats to capacity, Deficient Illness identity, Spiralling complexity.
Pearson et al 2021 (35)		Caregivers (S)+(F)	Thematic	The complex nature of informal caregiving, The barriers to caregiving, The facilitators of caregiving.
Brooman-White et al 2023 (36)	Critical Realism, Systems Thinking (I)	HCPs, Patients	Thematic	Working with complexity, Information transfer, Working relationships

**Table 4:** Line of argument

3rd order concepts	2nd order concepts	Definitions from 2^nd^ order data	Examples from 1^st^ order data	References
Difficult to diagnose (HCPs)	Uncertainty about clinical practice.	A lack of knowledge about diagnosis and management of HFpEF was evident across all professional groups, including many cardiologists. Issues around communicating the diagnosis of HF to patients and carers were highlighted. Generally, the lack of an evidence base for diagnosis and management of HFpEF meant that the guidelines for this condition were deemed to be less useful than those for LVSD… 9% of cardiologists remained unconvinced about the existence of HFpEF, which may have influenced the confidence of other clinicians when considering a diagnosis of HFpEF.	‘One cardiologist commented that they would like access to *“A crystal ball”* to inform HFpEF diagnosis, while a GP partner asked *“What is HFpEF?”*	Hancock et al (2014: 3)
	Diagnostic difficulty.	Some clinicians expressed concern that HFpEF may be overlooked in a system attuned to identifying patients with the more easily recognisable HFrEF.	‘I feel like the diagnosis probably comes later down the line because patients or clinicians are a bit thrown by “oh, they have a normal echo”, or “this doesn’t quite fit”.’ (General Practitioner)	Sowden et al (2020: e883)
		Patient descriptions of their route to diagnosis were often circuitous, for example, following several different specialty appointments. HFSNs described diagnosis being complicated by comorbidities, suggesting medical complexity may explain some of this discrepancy. Patients described managing multiple, diverse appointments frequently in the context of poor mobility, frailty and limited transportation options.	‘You will get a patient that is significantly breathless, but they may have underlying COPD [chronic obstructive pulmonary disease], and it’s trying to differentiate… it’s a real challenge.’ (Heart Failure Specialist Nurse 3)	Brooman-White et al (2023: 7)
	Difficulties in communicating the diagnosis.	Some GPs preferred to inform patients of a specific HFpEF label, others were less willing to do so for fear of upsetting patients by telling them that their heart was not pumping well or ‘failing’. This then contributed to difficulties in explaining what management could be offered.	‘I think the diagnosis of heart failure; whichever type of heart failure it is, it’s quite a hard conversation to have with patients because it’s quite a dramatic term, your heart is failing […] talking about that with patients can be difficult and I think there’s even more complexity to that with HFpEF patients because you say, “well, you have what we call heart failure, but actually it’s pumping alright, your heart, but what we know is these other things aren’t working as well, but we don’t actually have that much evidence about what will help, what will improve things.” (General Practitioner)	Hossain et al (2021:7)
Difficult to understand (Pts and Cs)	Poor communication	While some patients accepted ‘failure’ was a negative term, they emphasised the importance of understanding and making sense of their diagnosis, and many expressed a desire to know more.	‘One of the things which I find a big problem with the services you get from the hospital and the doctor, they don’t tell you enough.’(Patient)	Sowden et al (2020: e884)
		Patients generally valued being copied into letters. However, some felt that these lacked understandable information, particularly regarding medications and diagnoses, which could cause distress	‘It’s just the terminology they use really. I mean, if [patient1] received this letter on his own, he would think, what’s that? That sounds horrible. What does that mean?’ Patient 1 Carer	Brooman-White et al (2023: 8)
	Deficient Illness identity.	Once diagnosed, participants reported receiving information about HFpEF that was inaccurate. Clinical information provided in consultations affected patients’ belief about HFpEF, often leading to two misinterpretations. The first, ‘HFpEF is not that bad’ signalled to patients that there is no need to worry or make changes. The second, ‘nothing can be done’, related to the perceived lack of treatment options in HFpEF. Non-treatment was often understood as a lack of empathy, or lack of importance and sometimes undermined participants’ legitimacy as a patient with a need for treatment.	‘He [heart failure specialist nurse] said to me …you don’t know how lucky you are because … normally your heart condition, although you do have heart failure. is not to such a degree that you would normally merit a heart failure nurse.’ (Patient)	Forsyth et al (2023:5)
	Loss of identity.	[Patients] often compared their lives now to those before their illness, e.g. related to their occupational role or physical fitness. They were frustrated by how others (e.g. family, health care staff) now perceived them as individuals struggling with the constraints of their condition. Some yearned for the opportunity to demonstrate their more positive ‘former selves’, e.g. confident people with a purpose in life. One participant even expressed it may be better for himself and his family if he were dead	‘I don’t want to be here, and everybody says: That’s not fair to your wife or your kids. Wait a minute, I say, Really? This is unfair to my wife and my kids. My wife deserves to be taken away for the weekend. I can’t do that.’ (Patient)	Smith et al (2021:7)
Difficult to access support (Pts and Cs)	Multiple threats to capacity.	The effects of HFpEF, ageing, and multi-morbidity jointly conspired to create fear. Fears were multiple including exacerbation of symptoms, hospitalization, physical deterioration, loss of independence, falling, and fear of what other people might think if unable to control symptoms like breathlessness and urinary urgency. Fears affected capacity by reducing confidence and resilience to perform work (i.e. fear of falling preventing engagement in exercise) and function socially (i.e. reduced social engagement due to incontinence).	‘Sometimes I can get very anxious and then it takes my breath, that’s what worries me more than anything because I’ve got nobody here if I collapsed… if I’m on the floor, how do I get to my phone. It’s frightening.’ (Patient)	Forsyth et al (2023:5)
	Spiralling complexity.	Interaction with healthcare services rarely resulted in improvement in either capacity or workload, and the lack of significance afforded to HFpEF meant that participants were unable to elicit and affect change which contributed to the loss of ability.	‘Well, you can kind of plan or kind of adapt your life to know, or what’s going to happen in the future, or how to avoid making things worse, or… Any information like that, surely it’s going to help you adjust your life or not adjust your life to your condition.’ (Patient)	Forsyth (2023: 6)
	The complex nature of informal caregiving.	Carers frequently had a very active role in co-managing the health of the person they cared for. They often took responsibility for or provided practical assistance with organizing and attending medical appointments, managing medications, and supporting lifestyle changes like dietary adjustments.	‘My role in the family is to help him with his doctor’s appointments, hospital appointments and to make sure his tablets are fine, etc. You know, just that side of his life, to make sure the medical side is in order and that he’s keeping to his plan and what the doctor tells him to do.’ (Carer)	Pearson et al (2022: 4-5)
	The barriers to caregiving.	As HFpEF progressed, carers reported increasing levels of stress as the complexities of care escalated and the patient’s capacity to manage them independently deteriorated.	‘It’s been horrendous, because I’m his carer, and over the years he’s become quite frail…he doesn’t remember much, so I have to be there all the time.’ (Carer)	Pearson et al (2022: 5)
Made worse by organisational barriers (HCPs)	Management disparity.	Patients with HFpEF did not necessarily receive the same resources or opportunities as those with HFrEF, which was viewed as inequitable.	‘They [GPs] don’t receive QOF [Quality Outcomes Framework] points or payments for that particular group of patients [patients with HFpEF] so I don’t think we actively seek them out’ (Practice Nurse)	Sowden et al (2020: e885)
	Uncertainty in managing people with HFpEF.		‘…you know the ideal cardiological solution for them may not be the holistic solution for them…’ (General Practitioner)	Hossain et al (2021:8)
	Lack of coordination	While [patients and carers] could work to arrange appointments, monitor their health and take their medications, they identified a need for health services to respond to their requests for support. Many felt responsiveness was variable and described experiences of fragmented care and loss of trust.	‘And you go to the GP, and you don’t see the same GP… First you’ve got to get through the wall of the receptionists… I don’t feel confident… and to be honest, if I’m feeling that ill, I’m going to the hospital.’ (Patient 4)	Brooman-White et al (2023: 9)
		Clinicians echoed this, suggesting self-care needed to be properly supported by services and that poor resourcing and regional variation could limit this.	‘Health care isn’t really self-care, it’s self-care supported by the health service.’ (Commissioner)	
		Patients with accessible, relational continuity with a clinician reported a feeling of cohesion around their care, compared with patients who struggled to obtain this.	So even a nurse practitioner at the practice, whose keeping an overall view of what’s going on and what’s happening would help… there isn’t anybody… it just all seems very random. (Patient 2)	
	Discontinuity across the primary and secondary care interface.	[Some GPs] described uncertainty over where the responsibility for managing people with HF lay, and inadequate follow up in specialist care.	‘And there are some broader irritations in terms of the general practice, secondary care relationship in terms of who’s taking responsibility for some of the follow-up stuff […] it’s not always as clear as it should be, despite the new set of contracts that have come out of the, you know, the hospital should follow up hospital tests, they don’t always quite understand that.’ (General Practitioner)	Hossain et al (2021: 10)
		[A relational disconnect between primary care and specialist service] was coupled with service design and commissioning arrangements resulting in work being passed across the interface without shared understanding of work conditions. Requests perceived to be simple by secondary care were reported by GPs to be time and resource consuming. Clinicians felt that, in these circumstances, GPs functioned as an intermediary, providing logistical support rather than clinical expertise to patient care. However, the location of specialist services and patient mobility could limit alternative arrangements.	‘They’re really being used as a technical point, if you like, to get the blood tests done… because the GP is not necessarily involved in that episode of care…. So it kind of becomes fragmented.’ (Cardiologist 3)	Brooman-White et al (2023: 8)
	Shouldering a heavy workload.	Multiple co-morbid conditions were often accompanied by complex medication regimens that drove workload. The majority described polypharmacy which added to complexity and often resulted in negative effects such as potentially inappropriate medications or therapeutic competition.	‘[my carer] said to me why don’t you do a repeat prescription…well I can’t because I never know how much insulin I’m going to need and I’ve got a box up there and I do all my tablets once a week…it takes me about half an hour, you have to be really quiet to make sure you put the right ones in the right thing.’ (Patient)	Forsyth et al (2023: 4)
But HFpEF self-management can be optimised with support, when the burden is acknowledged, and when personalised goals and set and feedback provided.	Facilitation	Healthcare champions ranged from HF specialist nurses to consultants or general practitioners (GPs). Regardless of the person or role, they supported patients and their carers similarly through being empathetic, listening, active communication, and trouble shooting. For carers, these champions ensured they were involved in all aspects of care. Those who did not have a healthcare champion often described feeling lost or left to their own devices, uncertain where to get advice, or what to do in the face of deterioration.	‘whenever I feel I need to talk to him [GP], I always can and he’s always very good.’ (Patient) ‘but you’ve got no-one to talk to. […] but I can’t ask them [heart failure team] because they don’t know exactly what’s wrong. Nobody does I suppose really.’ (Carer)	Pearson et al (2022:5)
	Recognising and responding to emotion.	Patients and caregivers reported anger or low mood often related to their feelings of frustration associated with the limitations that HF imposed on their lifestyles. For six patients, the manual helped them to recognise their altered mood. Working with the facilitators enabled better management of these emotions, sometimes drawing on existing strategies, e.g. mindfulness or using new techniques, such as relaxation. Enabling patients to understand that these feelings were ‘normal’ under the circumstances allowed caregivers to support the patient’s psychological adjustment to their HF. Caregivers suggested that the intervention had reduced anxiety and improved mood, particularly in patients with elevated HADS scores.	‘I just feel once he started to understand more about heart failure, with the manual, that yes, he sort of -I don’t know, sort of maybe accepted it more… I think sometimes he sort of panics, thinking oh you know, should I be feeling this way? Whereas having the manual has, I think, sort of made him realise yes, this is normal for me to feel like this and be like this.’ (Caregiver)	Smith et al (2021: 6)
	Engagement with the REACH-HF intervention.	Participants confirmed that the REACH-HF manual provided information and reassurance: ‘offering something for every-one’. In combination with the Progress Tracker, this aided symptom monitoring and supported self-management. Patients’ and caregivers’ accounts again reinforced their need to understand how to manage HF by knowing what to look for in case of deterioration and what to do in an emergency. Through improved understanding, caregivers felt more confident in supporting the patients.	[Facilitator said] No, if you can’t do that what do you love doing? I say, I love walking. She went, Right, if you want to go out for a walk, let’s go out for a walk. (Patient)	Smith et al (2021: 6-7)
	Changes in health-related behaviours.		‘[The facilitator] was very helpful for me in so many different ways. Helping me to understand heart failure…she encouraged me to go out walking… Just the reassurance that things were better, that there was somebody there that was willing to, erm, say, well, okay, you’re doing well. Even just the smallest amount of encouragement. And ‘my husband always felt better after [the facilitator] went away. Because she felt…almost like a little security blanket, if you want to say. That somebody was there, somebody was asking.’ (Caregiver)	Smith et al (2021: 7-8)
	Monitoring and symptom tracking.	Proactive symptom monitoring… improved patients’ abilities to communicate with doctors to allow prescribing of appropriate treatment.	Every time [Facilitator] would come out she would start to look back through the stuff but she would go right to the front of the manual, not the manual the chart you call it, and would go through preceding weeks that she’d already covered (Patient)	Smith et al (2021:8)
	Unique caregiver views and experience.	There was a strong reluctance to be identified as ‘caregivers’, even when the ‘caregiver’ assisted the patient in activities such as washing and dressing. Caregivers regarded their caring role as ‘fluid’. Most described providing minimal physical assistance on a day-to-day basis with increased help when away from home or during episodes of patient’s illness. Caregivers also highlighted how balancing competing demands on their time (e.g. caring roles for other family members), or their own health status, could affect the support they were able to give.		Smith et al (2021: 8)

## Box 1 Explicit eligibility criteria

*Participants:* People with HFpEF/caregivers, or a discrete sample of people with HFpEF.

*Concept:* The experiences, beliefs about, and expectations of people living with and managing HFpEF/caregivers.

*Context:* Community dwelling adults, with experiences of management both in the community and as hospital outpatients (e.g. cardiac rehabilitation).

*Types of Sources:* Qualitative study designs (studies that have used qualitative methods of data collection (e.g. interviews, focus groups, or observations) *and* qualitative methods of analysis (e.g. thematic analysis, framework analysis). Systematic reviews that met the inclusion criteria and conference abstracts were also considered.

*Inclusion criteria:* Qualitative or mixed-methods study designs that capture the experience of people with HFpEF/caregivers, or the perspectives of HCPs who facilitate this management.

*Study design:* Qualitative methods of data collection (e.g. interviews, focus groups, or observations) *and* qualitative methods of analysis (e.g. thematic analysis, framework analysis); e.g. not numerical content analysis or statistical analysis.

*Study Outcomes:* Experience of people with HFpEF/caregivers. e.g. if a wider sample of patients with ‘heart failure’ there must be data specific to the HFpEF patient sub-sample. Experiences of health care professionals involved in facilitating self-management of HFpEF.

## Highlights box

- We applied an under-utilised research method (meta-study) to a poorly understood condition (HFpEF).
- We explored the current knowledge gap in understanding of the self-management experiences of people with HFpEF, their caregivers and the HCPs who support them.
- By working at the interface of secondary qualitative analysis (re-analysis of data) and qualitative synthesis (data integration) we identified how existing knowledge has been constructed, and our understanding of HFpEF management to date.
- The limited capacity of HCPs’ to support HFpEF patients’ and caregivers’ to develop effective self-management understanding and practice can lead to disempowerment and loss of control. Future research is required to specify and evaluate the skillset that HCPs require to provide individualised care that is tailored to the holistic needs of people self-managing HFpEF within the local care context.

## Reflective questions

1. What are the experiences of self-management for adults with HFpEF and their caregivers?
2. What are HCPs’ perspectives of facilitating self-management programmes for people with HFpEF and their caregivers?

## References

[1] Braunwald E (2015). The war against heart failure: the Lancet lecture. Lancet.

[2] National Institute for Health and Care Excellence (2018) (2018). Chronic heart failure in adults: diagnosis and management NICE guideline [NG106].

[3] Al-Mohammad A (2010). Chronic Heart Failure Guideline Development. Diagnosis and management of adults with chronic heart failure: Summary of updated NICE guidance. British Medical Journal.

[4] Lesyuk W, Kriza C, Kolominsky-Rabas P (2018). Cost-of-illness studies in heart failure: a systematic review. BMC Cardiovascular Disorders.

[5] Clark H, Rana R, Gow J, Pearson M, van der Touw T, Smart N (2022). Hospitalisation costs associated with heart failure with preserved ejection fraction (HFpEF): a systematic review. Heart Failure Reviews.

[6] Groenwegan A, Rutten FH, Mosterd A, Hoes AW (2022). Epidemiology of Heart Failure. European Journal of Heart Failure.

[7] McMurray J, Stewart S (2002). The burden of heart failure. European Heart Journal Supplements.

[8] Solano JP, Gomes B, Higginson IJ (2006). A comparison of symptom prevalence in far advanced cancer, AIDS, heart disease, chronic obstructive pulmonary disease and renal disease. Journal of Pain Symptom Management.

[9] Gallacher K, May C, Montori V, Mair F (2011). Understanding patient’s experiences of treatment burden in chronic heart failure: Using normalization process theory. Annals of Family Medicine.

[10] Jani B, Blane D, Browne D, Montori V, May C, Shippee N, Mair F (2013). Identifying treatment burden as an important concept for end of life care in those with advanced heart failure. Current Opinion in Supportive and Palliative Care.

[11] Ponikowski P, Voors AA, Anker SD, Bueno H, Cleland JGF, Coats AJS (2016). 2016 ESC guidelines for the diagnosis and treatment of acute and chronic heart failure. Eur J Heart Fail.

[12] Zakeri R, Cowie MR (2018). Heart failure with preserved ejection fraction: controversies, challenges and future directions. Heart.

[13] Taylor RS, Hayward C, Eyre V, Austin J, Davies R, Doherty P, Jolly K, Wingham J, Van Lingen R, Abraham C, Green C, on behalf of the REACH-HF investigators (2015). Clinical effectiveness and cost-effectiveness of the Rehabilitation Enablement in Chronic Heart Failure (REACH-HF) facilitated self-care rehabilitation intervention in heart failure patients and caregivers: rationale and protocol for a multicentre. randomised controlled trial. BMJ Open.

[14] Dalal HM, Taylor RS, Wingham J, Greaves CJ, Jolly K, Lang CC, Davis RC, Smith KM, Doherty PJ, Miles J, van Lingen R (2021). A facilitated home-based cardiac rehabilitation intervention for people with heart failure and their caregivers: a research programme including the REACH-HF RCT. Southampton (UK): NIHR Journals Library.

[15] Frost J, Wingham J, Britten N, Greaves C, Abraham C, Warren F, Jolly K, Doherty P, Miles J, Singh S, Paul K (2019). Home-based rehabilitation for heart failure with reduced ejection fraction: mixed methods process evaluation of the REACH-HF multicentre randomised controlled trial. BMJ Open.

[16] Barnett-Page E, Thomas J (2009). Methods for the synthesis of qualitative research: a critical review. BMC Medical Research Methodology.

[17] Ritzer G (1991). Metatheorizing in Sociology.

[18] Zhao S (1991). Meta-theory, meta-method, meta-data-analysis: What, why, and how?. Sociological Perspectives.

[19] Paterson B, Thorne S, Canam C, Jillings C (2001). Meta-Study of Qualitative Health Research. A Practical Guide to Meta-Analysis and Meta-Synthesis.

[20] Thorne S, Paterson B, Acorn S, Canam C, Joachim G, Jillings C (2002). Chronic illness experience: insights from a metastudy. Qual Health Res.

[21] Tong A, Flemming K, McInnes E, Oliver S, Craig J (2012). Enhancing transparency in reporting the synthesis of qualitative research: ENTREQ. BMC Medical Research Methodology.

[22] France EF, Cunningham M, Ring N, Uny I, Duncan EA, Jepson RG, Maxwell M, Roberts RJ, Turley RL, Booth A, Britten N (2019). Improving reporting of meta-ethnography: the eMERGe reporting guidance. BMC Medical Research Methodology.

[23] Cooke A, Smith D, Booth A (2012). Beyond PICO: the SPIDER tool for qualitative evidence synthesis. Qual Health Res.

[24] Wong SS, Wilczynski NL, Haynes RB, Hedges Team (2004). Developing optimal search strategies for detecting clinically relevant qualitative studies in MEDLINE. Stud Health Technol Inform.

[25] Ouzzani M, Hammady H, Fedorowicz Z, Elmagarmid A (2016). Rayyan—a web and mobile app for systematic reviews. Systematic Reviews.

[26] Uddin S, Hossain L, Rasmussen K (2013). Network effects on scientific collaborations. PLoS ONE.

[27] Critical Appraisal Skills Programme (2018). CASP Qualitative Checklist.

[28] Barbour RS (2000). Checklists for improving rigour in qualitative research: a case of the tail wagging the dog?. Journal of Evaluation in Clinical Practice.

[29] Dixon-Woods M, Sutton A, Shaw R, Miller T, Smith J, Young B, Bonas S, Booth A, Jones D (2007). Appraising qualitative research for inclusion in systematic reviews: a quantitative and qualitative comparison of three methods. Journal of Health Service Research Policy.

[30] Hancock HC, Close H, Fuat A, Murphy JJ, Hungin AP, Mason JM (2014). Barriers to accurate diagnosis and effective management of heart failure have not changed in the past 10 years: a qualitative study and national survey. BMJ Open.

[31] Sowden E, Hossain M, Chew-Graham C, Blakeman T, Tierney S, Wellwood I, Rosa F, Deaton C (2020). Understanding the management of heart failure with preserved ejection fraction: a qualitative multiperspective study. Br J Gen Pract.

[32] Hossain MZ, Chew-Graham CA, Sowden E, Blakeman T, Wellwood I, Tierney S, Deaton C (2022). Challenges in the management of people with heart failure with preserved ejection fraction (HFpEF) in primary care: A qualitative study of general practitioner perspectives. Chronic Illn.

[33] Smith K, Lang C, Wingham J, Frost J, Greaves C, Abraham C, Warren FC, Coyle J, Jolly K, Miles J, Paul K (2021). Process evaluation of a randomised pilot trial of home-based rehabilitation compared to usual care in patients with heart failure with preserved ejection fraction and their caregiver’s. Pilot Feasibility Stud.

[34] Pearson CR, Forsyth F, Khair E, Sowden E, Borja Boluda S, Deaton C, Optimise HFpEF Investigators (2023). ‘Keeping the plates spinning’: a qualitative study of the complexity, barriers, and facilitators to caregiving in heart failure with preserved ejection fraction. European Journal of Cardiovascular Nursing.

[35] Forsyth F, Blakeman T, Burt J, Chew-Graham CA, Hossain M, Mant J, Sharpley J, Sowden E, Deaton C (2023). Cumulative complexity: a qualitative analysis of patients’ experiences of living with heart failure with preserved ejection fraction. European Journal of Cardiovascular Nursing.

[36] Brooman-White R, Blakeman T, McNab D, Deaton C (2023). Informing understanding of coordination of care for patients with heart failure with preserved ejection fraction: a secondary qualitative analysis. BMJ Qual Saf.

[37] Moustakas C (1994). Phenomenological research methods.

[38] Husserl E (1970). The crisis of European sciences and transcendental phenomenology.

[39] Fuat A, Hungin APS, Murphy JJ (2003). Barriers to accurate diagnosis and effective management of heart failure in primary care: Qualitative study. British Medical Journal.

[40] Forsyth F, Mant J, Taylor CJ, Hobbs FR, Chew-Graham CA, Blakeman T, Sowden E, Long A, Hossain MZ, Edwards D, Deaton C (2019). Optimising Management of Patients with Heart Failure with Preserved Ejection Fraction in Primary Care (OPTIMISE-HFpEF): rationale and protocol for a multi-method study. BJGP Open.

[41] Heaton J (2004). Reworking Qualitative Data.

[42] May C, Finch T, Rapley T, Nilsen P, Birken S (2020). Handbook of Implementation Science.

[43] Shippee ND, Shah ND, May CR, Mair FS, Montori VM (2012). Cumulative complexity: a functional, patient-centered model of patient complexity can improve research and practice. J Clin Epidemiol.

[44] Sharpley J, Sharpley A (2023). The key to managing HFpEF? Fun, exercise, diet, and sun. European Journal of Cardiovascular Nursing.

[45] McNab D, McKay J, Shorrock S (2020). Development and application of ‘systems thinking’ principles for quality improvement. BMJ Open Qual.

[46] Thorne S, Morse J (1994). Critical Issues in Qualitative Research Methodology.

[47] Heaton J, Alasuutari P, Bickman L, Brannen J (2008). The Sage Handbook of Social Research Methods.

[48] Tong A, Sainsbury P, Craig J (2007). Consolidated criteria for reporting qualitative research (COREQ): a 32-item checklist for interviews and focus groups. International Journal for Quality in Health Care.

[49] Ritzer G (2008). The McDonaldization of society.

[50] Noblit GW, Hare RD (1988). Meta-ethnography: Synthesizing qualitative studies.

[51] Kalogirou F, Forsyth F, Kyriakou M, Mantle R, Deaton C (2020). Heart failure disease management: a systematic review of effectiveness in heart failure with preserved ejection fraction. ESC Heart Fail.

[52] Jaarsma T, Hill L, Bayes-Genis A, La Rocca HB, Castiello T, Čelutkienė J, Marques-Sule E, Plymen CM, Piper SE, Riegel B, Rutten FH (2021). Self-care of heart failure patients: practical management recommendations from the Heart Failure Association of the European Society of Cardiology. European Journal of Heart Failure.

[53] McDonagh TA, Metra M, Adamo M, Gardner RS, Baumbach A, Böhm M, Burri H, Butler J, Čelutkienė J, Chioncel O, Cleland JGF (2021). 2021 ESC Guidelines for the diagnosis and treatment of acute and chronic heart failure. European Heart Journal.

[54] Landau D, Phillips S (2023). Measures to improve the interface between primary and secondary care.

[55] Skivington K, Matthews L, Simpson SA, Craig P, Baird J, Blazeby JM (2021). A new framework for developing and evaluating complex interventions: update of Medical Research Council guidance. British Medical Journal.

[56] Clark A (2013). What are the components of complex interventions in healthcare? Theorizing approaches to parts, powers and the whole intervention. Social Science and Medicine.

[57] Allana S, Clark A (2018). Applying meta-theory to qualitative and mixed methods research: A discussion of critical realism and heart failure disease management interventions. International Journal of Qualitative Methods.

[58] Thompson D, Clark A Heart failure disease management interventions: time for reappraisal. European Journal of Heart Failure.

[59] Ziebland S, Hunt K (2014). Using secondary analysis of qualitative data of patient experiences of health care to inform health services research and policy. Journal of Health Services Research and Policy.

[60] Long-Sutehall T, Sque M, Addington-Hall J (2011). Secondary analysis of qualitative data: a valuable method for exploring sensitive issues with an elusive population?. Journal of Research in Nursing.

[61] Estabrooks C, Field P, Morse J (1994). Aggregating Qualitative Findings: An Approach to Theory Development. Qualitative Health Research.

[62] Turner K, Percival J, Kessler D, Donovan J (2018). Synthesizing Qualitative Data Sets to Improve the Design of Trials and Complex Health Interventions: A Worked Example. Qualitative Health Research.

[63] Chalmers I, Bracken M, Djulbegovic B, Garattini S, Grant J, Gülmezoglu A, Howells D, Ioannidis J, Oliver S (2014). How to increase value and reduce waste when research priorities are set. The Lancet.

