# Supplementary material for: Understanding the self-management experiences of people with heart failure with preserved ejection fraction (HFpEF), their caregivers and the health care professionals who support them: Systematic review and qualitative meta-study
Source: Br J Card Nurs. Author manuscript; Available in PMC 2025 Jul 7. (PMC7617877; doi:10.12968/bjca.2024.0084)
Supplement: Supplementary Material [file EMS205720-supplement-Supplementary_Material.pdf]

Supplementary File 1: People with HFpEF and their caregivers

Ovid MEDLINE(R) and In-Process, In-Data-Review & Other Non-Indexed Citations

1	Heart Failure, Diastolic/
2	preserved ejection fraction.ab,ti,tw.
3	Heart Failure/
4	Heart failure.ab,ti,tw.
5	HFpEF.ab,ti,tw.
6	1 or 2 or 3 or 4 or 5
7	Caregivers/
8	(partner or partners or families or familial or family or care giver* or caregiver* or carer*).ab,ti,tw.
9	7 or 8
10	(rehabilit* or exercise* or (exercise adj therapy)).ab,ti,tw.
11	Rehabilitation/ or Exercise/ or Exercise therapy/
12	(management or self-management or self care).ab,ti,tw.
13	10 or 11 or 12
14	6 and 9 and 13

Embase

1	diastolic heart failure/
2	preserved ejection fraction.ab,ti,tw.
3	Heart Failure/
4	(Heart adj1 failure).ab,ti.
5	HFpEF.ab,ti,tw.
6	1 or 2 or 3 or 4 or 5
7	caregiver/
8	(partner or partners or families or familial or family or care giver* or caregiver* or carer*).ab,ti,tw.
9	7 or 8
10	(rehabilit* or exercise* or (exercise adj therapy)).ab,ti,tw.
11	Rehabilitation/ or Exercise/ or cardiac heart rehabilitation/
12	(management or self-management or self-care).ab,ti,tw.
13	10 or 11 or 12
14	6 and 9 and 13

CINAHL- EBSCO

#	**Query**
S22	S5 AND S8 AND S14
S14	S9 OR S10 OR S11 OR S12 OR S13
S13	TI (management or "self-management" or "self care") OR AB (management or "self-management" or "self care")
S12	(MH "Rehabilitation, Cardiac")
S11	(MH "Exercise") OR (MH "Therapeutic Exercise")

S10	(MH "Rehabilitation")
S9	TI (rehabilit* or exercise* or (exercise N1 therapy)) OR AB (rehabilit* or exercise* or (exercise N1 therapy))
S8	S6 OR S7
S7	TI (partner or partners or families or familial or family or care giver* or caregiver* or carer*) OR AB (partner or partners or families or familial or family or care giver* or caregiver* or carer*)
S6	(MH "Caregivers")
S5	S1 OR S2 OR S3 OR S4
S4	TI HFpEF OR AB HFpEF
S3	TI (Heart N1 failure) OR AB (Heart N1 failure)
S2	TI "preserved ejection fraction" OR AB "preserved ejection fraction"
S1	(MH "Heart Failure")

#	**Query**
S13	S5 AND S8 AND S18
S12	TI (management or "self-management" or "self care") OR AB (management or "self-management" or "self care")
S11	(DE "Exercise")
S10	(DE "Rehabilitation")
S9	TI (rehabilit* or exercise* or (exercise N1 therapy)) OR AB (rehabilit* or exercise* or (exercise N1 therapy))
S8	S6 OR S7
S7	TI (partner or partners or families or familial or family or care giver* or caregiver* or carer*) OR AB (partner or partners or families or familial or family or care giver* or caregiver* or carer*)
S6	(DE "Caregivers")
S5	S1 OR S2 OR S3 OR S4
S4	TI HFpEF OR AB HFpEF
S3	TI (Heart N1 failure) OR AB (Heart N1 failure)
S2	TI "preserved ejection fraction" OR AB "preserved ejection fraction"
S1	DE "Heart Disorders"

Searches in Web of Science; SCOPUS using the same combination of keywords, text word

Supplementary File 2: Healthcare professionals

1	Heart Failure, Diastolic/
2	HFpEF.ab,ti,tw.
3	(rehabilit* or exercise* or (exercise adj therapy)).ab,ti,tw.
4	Rehabilitation/ or Exercise/ or Exercise therapy/
5	(management or self-management or self care).ab,ti,tw.
6	(nurse or nurses or GPs or general practitioner* or doctor or doctors or Physician* or Health professional* or health care worker* or Physiotherapist* or facilitator* or therapists).ab,ti,tw.
7	1 or 2
8	3 or 4 or 5
9	6 and 7 and 8
